# Novel Strategies for Preventing Fungal Infections—Outline

**DOI:** 10.3390/pathogens14020126

**Published:** 2025-02-01

**Authors:** Damilola J. Agbadamashi, Claire L. Price

**Affiliations:** Institute of Life Science, Swansea University Medical School, Swansea SA2 8PP, UK

**Keywords:** fungal infections, antifungal resistance, immunocompromised populations, antifungal therapies, vaccines, antifungal peptides, nanotechnology, probiotics, immunotherapy, omics technologies, global health, fungal prevention, innovative treatments, fungal biology

## Abstract

Fungal infections are a significant global health challenge, causing approximately 3.8 million deaths annually, with immunocompromised populations particularly at risk. Traditional antifungal therapies, including azoles, echinocandins, and polyenes, face limitations due to rising antifungal resistance, toxicity, and inadequate treatment options. This review explores innovative strategies for preventing and managing fungal infections, such as vaccines, antifungal peptides, nanotechnology, probiotics, and immunotherapy. Vaccines offer promising avenues for long-term protection, despite difficulties in their development due to fungal complexity and immune evasion mechanisms. Antifungal peptides provide a novel class of agents with broad-spectrum activity and reduced resistance risk, whilst nanotechnology enables targeted, effective drug delivery systems. Probiotics show potential in preventing fungal infections, particularly vulvovaginal candidiasis, by maintaining microbial balance. Immunotherapy leverages immune system modulation to enhance antifungal defenses, and omics technologies deliver comprehensive insights into fungal biology, paving the way for novel therapeutic and vaccine targets. While these approaches hold immense promise, challenges such as cost, accessibility, and translational barriers remain. A coordinated effort among researchers, clinicians, and policymakers is critical to advancing these strategies and addressing the global burden of fungal infections effectively.

## 1. Introduction

Fungal infections pose a significant global health burden, with an estimated 6.5 million severe cases annually, leading to approximately 3.8 million deaths [1]. Both superficial and invasive infections are particularly dangerous for immunocompromised individuals, including those with HIV/AIDS, cancer, organ transplants, or critical illness [2,3]. Superficial infections, such as thrush and dermatophytosis, affect the skin and mucous membranes, while invasive infections compromise sterile body sites like the circulatory system, vital organs, and the central nervous system [4].

Traditional antifungal treatments include azoles, polyenes, and echinocandins, each targeting different fungal structures [5]. However, increasing antifungal resistance, driven by medical and agricultural overuse, has made infections harder to treat [6]. Notably, fungal pathogens, such as *Aspergillus fumigatus* and *Candida auris*, have developed resistance through mutations and multidrug tolerance, heightening the global health threat [7,8]. The clinical impact of resistance includes increased morbidity, mortality, and treatment costs, necessitating new therapeutic approaches [9].

Existing antifungal drugs also pose toxicity challenges. Polyenes, like amphotericin B, can cause nephrotoxicity, while azoles are linked to hepatotoxicity, and echinocandins may lead to mild liver toxicity [10,11]. These limitations have prompted research into novel preventive strategies, including vaccines, antifungal peptides, nanoparticles, probiotics, immunotherapy, and omics technologies [12,13]. This review explores and summarizes these emerging approaches as potential breakthroughs in combating fungal infections.

## 2. The Role of Vaccines in Preventing Fungal Infections

Vaccination is one of the most effective methods for preventing infectious diseases [14]. However, developing vaccines for fungal infections has proven more challenging than for bacterial or viral pathogens due to the complexity of fungal cell structures and the immune evasion strategies they employ by covering β-1,3-glucan and chitin with different molecules [15]. Effective vaccination against invasive fungal infections relies on inducing robust cell-mediated immunity, characterized by Th1 and Th17 responses. This enhances the host’s phagocytic capabilities, leading to improved fungal clearance. The search for suitable vaccine antigens extends beyond virulence factors, opening up a vast array of potential targets for investigation [16].

The development of effective fungal vaccines faces numerous hurdles due to diverse host vulnerabilities and varied mechanisms of fungal infection. A universal fungal vaccine is unlikely, as no single antigen can provide broad protection. Instead, targeted vaccine strategies will be necessary for each major fungal pathogen, accounting for unique host–fungus interactions and pathogenic processes [17]. The challenges of being unable to develop a universal fungal vaccine might not be unconnected to the fact that diverse antigens have been adopted in the creation of different fungal vaccines. However, it has been reported that the conservation of specific compounds within fungal cell walls and plasma membranes presents an opportunity for developing a broadly protective vaccine. Shared antigens among diverse fungal pathogens could potentially serve as a common target, enabling protection against various mycoses and possibly even diseases caused by distinct microorganisms [18]. For instance, a conjugate vaccine combining β-1,3-glucan with diphtheria toxoid has demonstrated efficacy against a range of fungal pathogens, conferring dual protection against aspergillosis and candidiasis [19].

In addition, vaccines targeting *Candida*, *Aspergillus*, and *Cryptococcus* species, which are among the most common causes of invasive fungal infections, are currently under development [20]. One promising approach involves the use of recombinant protein-based vaccines. These vaccines, which contain specific fungal antigens, stimulate the immune system to recognize and attack the fungal pathogen. For example, NDV-3A, a vaccine targeting *C. albicans* and *Staphylococcus aureus* (a bacterium, which is the causal agent of a variety of skin infections, respiratory infections, and food poisoning), has shown promising results in preclinical studies and early-phase clinical trials [21]. Another promising candidate is the *Cryptococcus neoformans* vaccine, which has shown efficacy in animal models [22] and may soon be evaluated for human use. Although fungal vaccines are not yet widely available, these advances represent a significant step toward preventing fungal infections in high-risk populations. Also, researchers are actively exploring alternative targets, such as Als3p, a key component of *C. albicans*’s cell wall, with encouraging preliminary results [23]. These innovative approaches pave the way for developing more efficacious fungal vaccines. Further information on vaccines can be found in the review by Riveria et al., 2022 [24].

Despite significant challenges, the development of fungal vaccines is advancing, offering hope for more effective prevention strategies. The complexity of fungal cell structures and immune evasion tactics has made vaccine development difficult, but promising candidates are showing encouraging results. Targeting shared fungal antigens, like β-1,3-glucan, could lead to broader protection against multiple pathogens. However, due to the diversity of fungal infections and host responses, a universal vaccine remains unlikely. Future research should focus on optimizing antigen selection, improving immune response strategies, and integrating these vaccines into clinical practice, particularly for high-risk populations. Continued innovation and investment in fungal immunization will be crucial to overcoming current limitations and reducing the global burden of fungal infections.

## 3. Antifungal Peptides: A Novel Class of Antifungal Agents

Antifungal peptides (AFPs) are short chains of amino acids that possess broad-spectrum antifungal activity [25]. These peptides, often derived from natural sources such as plants, animals, and microorganisms, have garnered attention due to their ability to disrupt fungal cell membranes and inhibit fungal growth [26,27]. AFPs represent a novel class of antifungal agents that could complement or replace traditional antifungal drugs, especially in the context of increasing drug resistance [25]. The rapid evolution and adaptability of fungi pose significant challenges to antifungal treatments. However, AFPs target cell membranes, which evolve more slowly, rendering them effective against fungal pathogens [28]. This multifaceted mechanism minimizes the risk of resistance emergence in target pathogens [29]. However, prudent AFP usage is crucial to prevent accelerated resistance development, as observed with conventional antimicrobials [28].

Nonetheless, AFPs offer several advantages over conventional antifungal agents, stemming from their distinctive modes of action and molecular specificity. Their capacity to bind multiple microbial targets reduces the potential for resistance development [30]. AFPs also exhibit low cytotoxicity due to their specific interaction with conserved fungal targets, such as glucosylceramide and enzymes essential for ergosterol biosynthesis. This selective binding reduces harm to mammalian cells [30]. AFPs have a complex role in therapy, with Host Defense Peptides (HDPs), such as defensins and cathelicidins, demonstrating angiogenic, immunomodulatory, and anti-inflammatory properties, in addition to recruiting adaptive immune cells, thereby exhibiting a multifaceted therapeutic profile [31,32].

Several AFPs have demonstrated potent antifungal activity against various fungal pathogens. For example, defensins, a family of peptides produced by plants and animals, exhibit antifungal properties by disrupting fungal cell walls and inhibiting spore germination [33]. Another promising group of AFPs includes histatins, salivary peptides that have shown activity against *C. albicans* [34]. Also, cathelicidins, such as LL-37, exhibit potent anti-adhesive activity against *C. albicans* in a murine urinary tract model, resulting in a substantial reduction (over 70%) in yeast attachment to bladder tissue [35]. These peptides offer a potential alternative to conventional antifungals, with the added benefits of low toxicity and reduced risk of developing resistance.

Although AFPs holds promise for combating infections resistant to conventional treatments, a critical knowledge gap persists regarding the interplay between their molecular structure and biological activity, thereby impeding accelerated translation into therapeutic applications [25]. This explains why, to date, only a small number of antifungal peptides have entered clinical trials, including nikkomycin Z, aureobasidin A, and VL-2397, which hold significant promise for addressing the urgent need for novel antifungal treatments [4]. Nevertheless, a coordinated approach among stakeholders—regulatory bodies, researchers, and industry leaders—is crucial for accelerating the development of peptide-based antifungal solutions. Peptides’ potent antifungal activity, stability across various temperatures and pH levels, and resistance to proteolysis make them attractive candidates for clinical therapeutics, warranting further investigation and collaboration [26,36].

AFPs represent a promising alternative to conventional antifungal treatments, offering broad-spectrum activity, low toxicity, and a reduced likelihood of resistance development. Their ability to target fungal cell membranes and essential biosynthetic pathways makes them effective against drug-resistant pathogens. Peptides such as defensins, histatins, and cathelicidins have demonstrated potent antifungal properties, highlighting their therapeutic potential. However, challenges remain, particularly in understanding the relationship between their molecular structure and biological activity, which has slowed clinical translation. While a few AFPs, including nikkomycin Z and VL-2397, have entered clinical trials, further research, regulatory support, and industry collaboration are necessary to advance peptide-based antifungal solutions. With continuous innovation and strategic investment, AFPs could play a critical role in addressing the growing threat of antifungal resistance and expanding treatment options for fungal infections.

## 4. Nanoparticles and Nanotechnology-Based Therapies

The current treatments for systemic fungal infections face significant challenges due to existing antifungals’ inadequate distribution, efficacy, and specificity, as well as their potential for severe adverse effects [37]. However, nanotechnology offers a promising solution, enabling the development of targeted and controlled drug delivery systems through nanoparticles (NPs). This innovative approach can enhance the effectiveness of fungal infection treatments while minimizing harm to patients’ overall well-being [38]. Thus, nanotechnology-based therapies are gaining attention for their potential to revolutionize the prevention and treatment of fungal infections [39]. Various studies have shown that nanoparticles exhibit reduced side effects, enhanced targeting of infection sites, do not lead to drug resistance, improve the stability and solubility of antifungal agents, and boost overall effectiveness [40,41,42]. Moreover, nanoparticles can be tailored with precise surface modifications, enabling selective recognition and binding to diseased cells, thereby enhancing therapeutic precision, minimizing harm to healthy cells, and optimizing drug performance [43].

Various types of nanoparticles have been explored for antifungal purposes, including liposomes, metallic nanoparticles, and polymeric nanoparticles. Liposomal formulations, such as liposomal amphotericin B, have already been approved for clinical use and have demonstrated enhanced efficacy and reduced toxicity compared to conventional amphotericin B [44]. Metallic nanoparticles, such as silver and gold nanoparticles, have also shown promise due to their antifungal properties and ability to disrupt fungal biofilms [45,46]. A study demonstrated that the enhanced antifungal efficacy of biosynthesized silver nanoparticles (AgNPs) stabilized with sodium dodecyl sulfate (SDS) and reduced using ribose has significant inhibitory effects against *C. albicans* and *C. tropicalis* [47]. In addition, Fajar et al. (2019) [48] have shown that AgNPs inhibit *Aspergillus niger* growth by up to 70% and suppress *Cladosporium cladosporoides* growth by up to 90%. The antifungal activity of AgNPs exhibits a dose-dependent relationship, with increased concentrations minimizing fungal growth.

Silver nanoparticles have been found to exhibit significant antifungal properties against *A. niger*. They achieve this by preventing spore germination and biofilm formation. When combined with simvastatin, a statin that disrupts fungal cell membrane integrity, the antifungal effect is enhanced. This synergy is likely due to the increased susceptibility of fungal cells to nanoparticle entry [49]. Silver nanoparticles have shown potent antifungal activity against *Trichophyton mentagrophytes* clinical isolates and *C. albicans* [50]. Nanotechnology-based strategies not only provide more effective drug delivery systems but also offer new avenues for developing antifungal therapies that could prevent infection at early stages [51]. Despite the promise of emerging therapies, significant uncertainties surround their effectiveness and safety in humans. Some studies lack rigorous clinical trials, and practical limitations, such as cumbersome administration methods or high production costs, may impede their adoption as viable alternatives to established treatments [52,53,54].

Nanotechnology presents a promising frontier in antifungal therapy, addressing key challenges associated with conventional treatments, such as poor drug distribution, toxicity, and resistance. Nanoparticles, including liposomes, metallic nanoparticles, and polymeric formulations, have demonstrated enhanced efficacy, targeted delivery, and reduced side effects. Liposomal amphotericin B is already in clinical use, while silver nanoparticles exhibit strong antifungal properties, disrupting biofilms and inhibiting fungal growth. Additionally, synergistic approaches, such as combining silver nanoparticles with simvastatin, further enhance the antifungal efficacy. However, despite these advancements, challenges remain, including high production costs, potential safety concerns, and the need for more rigorous clinical trials. Moving forward, continued research, regulatory validations, and industry collaborations will be crucial for translating nanotechnology-based antifungal strategies into viable clinical treatments, ultimately improving patient outcomes and expanding therapeutic options.

## 5. Probiotics and Their Role in Fungal Infection Prevention

Probiotics, which are live microorganisms that confer health benefits when consumed in sufficient amounts, have gained popularity for their role in maintaining a balanced microbiota and preventing infections [55]. While probiotics are commonly associated with bacterial infections, recent studies have demonstrated their potential in preventing fungal infections, particularly those caused by *Candida* species [56,57]. Research suggests that probiotics, when used as adjunctive treatment, may enhance short-term outcomes for fungal infections, leading to improved clinical and mycological resolution rates and reduced relapse rates within a one-month period; however, their effectiveness as a long-term solution remains uncertain [58].

Studies have shown that probiotics offer protective benefits against fungal infections, particularly vulvovaginal candidiasis (VVC). Both oral and vaginal probiotic administration can significantly alleviate symptoms of discharge and itching/irritation [59]. Furthermore, probiotics have been shown to substantially reduce VVC recurrence rates compared to placebo, with notable differences observed over extended follow-up periods [57,58]. Notably, probiotic-treated individuals exhibited improved symptom management and reduced recurrence rates at both 3- and 6-month follow-ups, underscoring the potential of *Lactobacilli*-based mixtures as a safe and effective adjunctive therapy for managing recurrent VVC [58]. Probiotics have been found to exhibit strong anti-biofilm properties during early fungal biofilm formation. However, their inhibitory effects are substantially reduced against biofilms in later stages of growth [60,61]. Therefore, more research is needed to fully understand the mechanisms underlying probiotic-mediated fungal inhibition, because probiotics represent a promising, natural strategy for preventing fungal infections, particularly in immunocompromised individuals.

Inconsistent results from several studies have sparked controversy regarding the efficacy of probiotics in addressing VVC, potentially attributed to variations in strain specificity, administration methods, and treatment regimens [62,63]. It has been argued that the effectiveness of probiotics in managing VVC varies, and it depends on the patient’s specific condition, whether it is an acute episode, recurrent infection, or heightened susceptibility to infection [64].

Probiotics offer a promising, natural approach to preventing and managing fungal infections, particularly *Candida*-related conditions, such as VVC. While probiotics may serve as a valuable adjunctive therapy, further research is needed to better understand their mechanisms of action and optimize their clinical applications. Moving forward, standardized probiotic formulations and well-designed clinical trials will be essential to establish probiotics as a reliable antifungal intervention, particularly for immunocompromised individuals and those prone to recurrent infections.

## 6. Immunotherapy: Harnessing the Immune System to Combat Fungal Infections

Immunotherapy, which involves modulating the immune system to enhance its ability to fight infections, has emerged as a novel approach for preventing fungal infections [65]. Fungal pathogens can evade the immune system by masking their antigens or suppressing immune responses, making it challenging for the body to mount an effective defense [15]. Immunotherapy aims to overcome these challenges by boosting the immune system’s capacity to recognize and eliminate fungal pathogens [66]. Immunity against fungal infections primarily relies on the activation of cellular immune responses, mediated by CD4^+^ T-helper cells. Specifically, Th1 and Th17 responses trigger the release of pro-inflammatory cytokines, including IL-12, IL-17A, IFN-γ, GM-CSF, and TNF-α, which mobilize various immune cells, such as neutrophils, macrophages, and dendritic cells [67]. Conversely, fungal infection progression is associated with a shift from Th1-type responses to Th2-mediated responses, characterized by CD4^+^ T-helper cells producing cytokines like IL-4, IL-5, and IL-10 [68].

Several immunotherapeutic strategies are currently being explored for fungal infection prevention, and preclinical studies have consistently demonstrated that cytokines significantly strengthen the immune system’s ability to combat fungal infections, largely by optimizing the performance of phagocytic cells. Extensive research in both human and murine models has unequivocally demonstrated that IL-17 plays a crucial and remarkably specialized role in safeguarding against *C. albicans* [69]. Although IL-2 and IL-12 have shown promise therapeutically, their clinical utility is limited due to systemic toxicity issues. In contrast, IL-23, a cytokine closely related to IL-12, has emerged as a key player in the immune response to chronic fungal infections [70].

Another approach involves the use of immune checkpoint inhibitors, which block inhibitory signals that dampen the immune response, thereby enhancing the activity of immune cells against fungal pathogens [71]. Another strategy is adoptive T-cell therapy, which involves transferring T cells from a healthy donor to an immunocompromised patient to boost their immune defense against fungal infections [72]. In the context of fungal immunity, T cells play a dual role as modulators and effectors. The Th1 response, initiated by TLR4 signaling, drives the production of IFN-γ and TNF-α, which are essential for fungal pathogen clearance. In contrast, TLR2-mediated Th2 responses generate anti-inflammatory cytokines, including IL-4 and IL-10, which regulate inflammation but compromise fungal resistance. Recently, Th17 cells have gained recognition for their crucial involvement in mucosal immunity against *Candida* [73]. Although these therapies are still in experimental stages, they hold great promise for preventing fungal infections, especially in individuals with weakened immune systems.

Immunotherapy represents a promising strategy for preventing fungal infections by enhancing the immune system’s ability to recognize and eliminate fungal pathogens. Emerging approaches, including cytokine therapy, immune checkpoint inhibitors, and adoptive T-cell therapy, show potential in boosting immune defense, particularly in immunocompromised individuals. However, challenges such as systemic toxicity and the complexity of immune regulation remain significant barriers to clinical application. Moving forward, further research and clinical trials are needed to refine immunotherapeutic strategies, minimize adverse effects, and develop targeted interventions. With continued advancements, immunotherapy could become a valuable tool in combating fungal infections, particularly for high-risk populations.

## 7. Omics Technologies for Fungal Infection Prevention

The advent of omics technologies, such as genomics, proteomics, and metabolomics, has revolutionized our understanding of fungal biology and opened new possibilities for preventing fungal infections [74,75]. These technologies enable researchers to study fungal pathogens at the molecular level, identifying potential targets for new antifungal therapies and vaccines [76]. For example, genomics has provided valuable insights into the genetic makeup of fungal pathogens, allowing for the identification of virulence factors and drug resistance genes [77]. Proteomics has facilitated the discovery of fungal proteins involved in host–pathogen interactions, which could serve as targets for vaccine development or therapeutic interventions [78]. Additionally, metabolomics has shed light on the metabolic pathways that fungi rely on for survival, offering potential targets for novel antifungal drugs [79]. By integrating omics data, researchers can develop more effective strategies for preventing fungal infections and overcoming the challenges posed by drug-resistant pathogens.

A comprehensive metabolomic analysis of antifungal resistance in *C. albicans* was conducted using a combination of cutting-edge mass spectrometry techniques, including ultra-high-performance liquid chromatography coupled with quadrupole time-of-flight mass spectrometry (UHPLC-Q-TOF/MS) and hydrophilic interaction liquid chromatography–mass spectrometry (HILIC-MS) for targeted phospholipid metabolism. This integrated approach revealed a wide array of metabolite biomarkers associated with drug stress response and resistance mechanisms, highlighting alterations in amino acid, sphingolipid, and phospholipid metabolic pathways [80]. In addition, the integration of transcriptomics and epigenomics has significantly enhanced the comprehension of complex cellular dynamics. A growing body of evidence indicates that immune cells exhibit metabolic adaptability, tailoring their responses to meet distinct defensive needs through the coordinated action of epigenetic regulators and metabolic networks [81,82].

Recent research has leveraged omics technologies to elucidate specific immune responses elicited by fungal infections. For example, de Jesús-Gil et al. (2021) [83] discovered that *C. albicans* triggers IL-17-mediated immunity, particularly in psoriasis patients, highlighting the critical role of IL-17 in inflammatory processes. This finding has significant implications for identifying therapeutic targets. Conversely, Stuehler et al. (2015) [84] investigated Th1-mediated immunity against *A. fumigatus* antigens, a fungus notorious for causing respiratory infections in immunocompromised individuals. The ability to pinpoint these precise immune reactions is vital for developing tailored and effective treatments.

The integration of cutting-edge omics technologies, including genomics, transcriptomics, proteomics, and metabolomics, provides a holistic understanding of the complex interactions between fungi and the immune system [75]. By leveraging these approaches, researchers can identify novel fungal antigens and immune targets, paving the way for the development of innovative vaccines and immunotherapies [85]. Furthermore, these insights can inform the design of adoptive T-cell transfer therapies, which involve genetically modifying immune cells to enhance their antifungal efficacy, offering new hope for combating fungal infections [86].

Despite the prospect of omics technologies, their adoption in fungal prevention faces several challenges. One key issue is the complexity of fungal genomes, which makes data interpretation difficult. High-throughput techniques, like genomics, proteomics, and metabolomics, generate vast amounts of data that require advanced bioinformatics tools for analysis, creating a barrier for widespread use [74,75]. Additionally, the cost of implementing omics technologies is high, limiting access for many researchers, particularly in low-resource settings [87]. Ethical concerns regarding data privacy and the need for standardization across platforms also hinder progress [88].

## 8. Conclusions

The rise in antifungal resistance and the prevalence of fungal infections underscore the urgent need for innovative approaches for prevention and treatment. Whilst conventional antifungal therapies can be effective, we are observing increasing issues with resistance, toxicity, and limited therapeutic options, particularly in immunocompromised patients. This underlines the need for a multifaceted strategy that integrates novel preventive measures and therapeutic interventions.

Advanced approaches such as vaccines, antifungal peptides, nanotechnology, probiotics, immunotherapy, and omics technologies offer promising pathways to address these challenges. Each strategy brings unique advantages and challenges. Addressing these challenges will require collaborative efforts among researchers, healthcare professionals, regulatory authorities, and industry stakeholders. By leveraging cutting-edge innovations alongside traditional therapies, we can improve outcomes for patients and mitigate the growing threat of fungal infections to global health.
